# Advanced therapies for the treatment of hemophilia: future perspectives

**DOI:** 10.1186/1750-1172-7-97

**Published:** 2012-12-13

**Authors:** Antonio Liras, Cristina Segovia, Aline S Gabán

**Affiliations:** 1 Department of Physiology, School of Biology, Complutense University of Madrid, and Cell Therapy and Regenerative Medicine Unit, La Paz University Hospital Health Research Institute-IdiPAZ, Madrid, Spain; 2 Royal Foundation “Victoria Eugenia” of Hemophilia, Madrid, Spain; 3 University for the Development of State and the Pantanal Region, Campo Grande, Brazil

**Keywords:** Advanced therapies, Gene therapy, Cell therapy, Hemophilia A, Hemophilia B

## Abstract

Monogenic diseases are ideal candidates for treatment by the emerging advanced therapies, which are capable of correcting alterations in protein expression that result from genetic mutation. In hemophilia A and B such alterations affect the activity of coagulation factors VIII and IX, respectively, and are responsible for the development of the disease. Advanced therapies may involve the replacement of a deficient gene by a healthy gene so that it generates a certain functional, structural or transport protein (gene therapy); the incorporation of a full array of healthy genes and proteins through perfusion or transplantation of healthy cells (cell therapy); or tissue transplantation and formation of healthy organs (tissue engineering). For their part, induced pluripotent stem cells have recently been shown to also play a significant role in the fields of cell therapy and tissue engineering. Hemophilia is optimally suited for advanced therapies owing to the fact that, as a monogenic condition, it does not require very high expression levels of a coagulation factor to reach moderate disease status. As a result, significant progress has been possible with respect to these kinds of strategies, especially in the fields of gene therapy (by using viral and non-viral vectors) and cell therapy (by means of several types of target cells). Thus, although still considered a rare disorder, hemophilia is now recognized as a condition amenable to gene therapy, which can be administered in the form of lentiviral and adeno-associated vectors applied to adult stem cells, autologous fibroblasts, platelets and hematopoietic stem cells; by means of non-viral vectors; or through the repair of mutations by chimeric oligonucleotides. In hemophilia, cell therapy approaches have been based mainly on transplantation of healthy cells (adult stem cells or induced pluripotent cell-derived progenitor cells) in order to restore alterations in coagulation factor expression.

## Introduction

Hemophilia is a recessive X-linked hereditary disorder caused by a deficiency of coagulation factor VIII (hemophilia A) or IX (hemophilia B). The disease is considered to be severe when factor levels are below 1% of normal values, moderate when they are between 1 and 5% and mild when levels range between 5% and 40% [1]. As the prevalence of the disease is 7.7/100,000, it is considered a rare hematologic disorder (Orpha number ORPHA448) (Table 1).

**Table 1:** **Classification (Orpha number), synonyms, prevalence and clinical description of hemophilia (Adapted from Orphanet portal [*http://www.orpha.net *])**

**Type of hemophilia**	**Orpha number**	**Synonyms**	**Prevalence**	**Clinical description**
Hemophilia (general)	448	Factor VIII or IX deficiency	7.7/100,000	Bleeding episodes.
Hemophilia A	98878	Factor VIII deficiency	1/6,000	Spontaneous or prolonged hemorrhages.
Hemophilia B	98879	Factor IX deficiency	1/30,000	Spontaneous or prolonged hemorrhages.
Mild hemophilia A	169808	Mild factor VIII deficiency	40% of all cases of hemophilia A	Small deficiency of factor VIII leading to abnormal bleeding as a result of minor injuries, or following surgery or tooth extraction. The biological activity of factor VIII is between 5 and 40%. Spontaneous hemorrhages do not occur.
Mild hemophilia B	169799	Mild factor IX deficiency	30% of all cases of hemophilia B	Small deficiency of factor IX leading to abnormal bleeding as a result of minor injuries, or following surgery or tooth extraction. The biological activity of factor IX is between 5 and 40%. Spontaneous hemorrhages do not occur.
Moderately severe hemophilia A	169805	Moderately severe factor VIII deficiency	20% of all cases of hemophilia A	Abnormal bleeding as a result of minor injuries, or following surgery or tooth extraction. The biological activity of factor VIII is between 1% and 5%. Spontaneous hemorrhages are rare.
Moderately severe hemophilia B	169796	Moderately severe factor IX deficiency	30% of all cases of hemophilia B	Abnormal bleeding as a result of minor injuries, or following surgery or tooth extraction. The biological activity of factor IX is between 1% and 5%. Spontaneous hemorrhages are rare.
Severe hemophilia A	169802	Severe factor VIII deficiency	40% of all cases of hemophilia A	Frequent spontaneous hemorrhage and abnormal bleeding as a result of minor injuries, or following surgery or tooth extraction. The biological activity of factor VIII is below 1%.
Severe hemophilia B	169793	Severe factor IX deficiency	40% of all cases of hemophilia B	Frequent spontaneous hemorrhage and abnormal bleeding as a result of minor injuries, or following surgery or tooth extraction. The biological activity of factor IX is below 1%.
Symptomatic form of hemophilia A in female carriers	177926	Hemophilia A carriers	Unknown(very rare)	In some women with mutations in the *F8* gene, encoding coagulation factor VIII. Symptoms include abnormal bleeding as a result of minor injuries, or following surgery or tooth extraction. Spontaneous hemorrhages may occur occasionally. The biological activity of factor VIII is below ~30%.
Symptomatic form of hemophilia B in female carriers	177929	Hemophilia B carriers	Unknown (very rare)	In some women with mutations in the *F9* gene, encoding coagulation factor IX. Symptoms include abnormal bleeding as a result of minor injuries, or following surgery or tooth extraction. Spontaneous hemorrhages may occur occasionally. The biological activity of factor IX is below ~30%.

As other medical conditions, hemophilia is a chronic disease associated with substantial healthcare and pharmaceutical costs which, in less advantaged countries, could result in disabling clinical conditions if not in a higher mortality rate. Contrary to other rare diseases, over the last few decades hemophilia has benefited from a greater understanding of the causes and mechanisms responsible for its development as well as of the molecular and physiological characteristics of the disease and its proper diagnostic and clinical management (Table 2). This has undoubtedly aided in the design of highly appropriate treatment schedules [1].

**Table 2:** Some molecular and physiological characteristics of hemophilia A and B

**Type of hemophilia**	**Deficient factor**	**Protein size (Kilodaltons)**	**DNA length (bp)**	**Half life (Hours)**	**Function in the coagulation cascade**	**Plasma concentration (ng/ml)**^**1,2**^		
						Mild hemophilia	Moderately Severe Hemophilia	Severe Hemophilia
Hemophilia A	VIII	280	7056	8-12	Activated by thrombin and activates to FX^3^	10-80 (5-40%)	2-10 (1-5%)	< 2 (<1%)
Hemophilia B	IX	68	1389	15-25	Activated by FXIa^4^, and activates to FX	500-2000 (5-40%)	50-500 (1-5%)	< 50 (<1%)

^1^Normal plasma level of coagulation factors: Factor VIII, 200 ng/mL; factor IX, 5000 ng/mL; ^2^One International Unit (IU) of factor VIII or IX, per kg of body weight, increases the activity of factor in plasma, 1% to 2% of normal level; ^3^FX, factor X; ^4^FXIa, factor XI activated.

The etiopathogenesis of the disease is related to different kinds of mutations (large deletions and insertions, inversions and point mutations) that occur in the gene expressing the deficient coagulation factor. The clinical characteristics of both types of hemophilia are very similar: spontaneous or traumatic hemorrhages, muscle hematomas, hemophilic arthropathy resulting from the articular damage caused by repetitive bleeding episodes in the target joints, or hemorrhages in the central nervous system (Table 1). In the absence of appropriate replacement treatment with exogenous coagulation factors, these manifestations of the disease can have disabling or even fatal consequences thus negatively impacting patients' quality of life and reducing their life expectancy [2]. Bleeding episodes may be spontaneous in the severe and (less so) in the moderate forms of the disease, with 70% of them being articular, 15% muscular and 15% visceral.

Diagnosis of hemophilia is aimed at identifying type of hemophilia and degree of involvement as well as detecting symptomatic or asymptomatic carriers of the disease, either obligate (daughters of hemophiliacs) or *de novo* (women by sporadic and spontaneous mutations). Diagnostic methods are based on the determination of coagulation factor levels in plasma and the detection of the mutation in the DNA extracted from peripheral blood leucocytes by means of direct or indirect genetic methods (detection of genetic polymorphism).

As hemophilia is a gender-related condition, genetic guidance is provided to couples on the basis of an odds analysis based on genealogy and coagulation factor data and a study of the mutation responsible for the disease. Moreover, either a prenatal diagnosis [3], —which can now be carried out non-invasively [4]—, or a pre-implantational diagnosis are provided [5]. In this respect, it is necessary to emphasize that access to prenatal or pre-implantation genetic diagnosis is not universally available but rather depends on the economic constraints imposed by different countries in accordance with their development status.

At present, patients with hemophilia benefit from optimized treatment schedules based on the intravenous systemic delivery of exogenous coagulation factors, either prophylactically or on demand protocols. The current policy in developed countries is in general to administer a prophylactic treatment (2 or 3 times a week) from early childhood into adulthood [2]. Such prophylactic protocols result in a marked improvement in patients' quality of life on account of the prevention of hemophilic arthropathy and other fatal manifestations of the disease as well as a reduction in the long-term costs of treatment because of a decrease in the need of surgical procedures such as arthrodesis, arthroplasty or synovectomy [6].

Conventional treatment of hemophilia [7,8] is currently based on the use of plasma-derived products —duly treated with heat and detergent to inactivate lipid-coated viruses [9]—, or the recently developed recombinant high-purity coagulation factor concentrates which do not contain proteins of human or animal origin [10,11]. Both kinds of factor boast high efficacy and safety profiles, at least for the inactivation-susceptible pathogens known to date. The choice of one product over the other is usually based on the clinical characteristics of the patient and on cost and availability considerations [12,13].

Now that infections by pathogenic viruses (VIH, HCV) that were common a few decades ago have been eradicated, the most distressing adverse effect observed when using both recombinant and plasma-derived products is the development of antibodies (inhibitors) against the perfused exogenous factors [14,15]. The appearance of inhibitors renders current treatment with factor concentrates inefficient, increasing morbidity and mortality, leading to the early onset of hemophilic arthropathy and disability and to a consequent reduction in patients' quality of life. Inhibitors also result in higher costs as treatment must be provided both for bleeding episodes and inhibitor eradication (immune tolerance induction). The incidence of inhibitors is around 30% in hemophilia A and 6% in hemophilia B [2].

The immunologic mechanism whereby these neutralizing antibodies are generated is highly complex and involves several messenger molecules (tumor necrosis factor, interleukins…), and cells (T-lymphocytes B-lymphocytes, macrophages …). They are directed at certain regions in the factor molecule that interact with other components of the coagulation cascade and, depending on their titre level and on whether they are transient or persistent, they will bring about greater or lesser alterations in the coagulation cascade. The causes that influence inhibitor development may be genetic —inherent in the patients themselves [14]—, such as ethnicity, familial history, type of mutation or certain changes in some of the genes involved in the immune response; or non-genetic —environmental [16]—, such as stimulation of the immune system by other antigens or the treatment regimen used (prophylactic vs. on demand). The influence of type of factor concentrate used (plasma-derived or recombinant) is still a subject for debated [17,18].

Short and medium-term perspectives for the treatment of hemophilia strongly rely on the current research efforts directed increasing the safety levels of (especially) plasma-derived factors with respect to the detection and subsequent inactivation of blood-borne pathogens in donors, such as prions and other potential emerging agents [19-21]. It is also important to enhance the efficiency of recombinant factors increasing their half-life (by PEGylating the factor molecule or using fusion proteins, both for factor VIII and factor IX [22-25]). These studies are now in progress and no definite statements can be made about the safety of long-acting products as none of them have yet been authorized for clinical use. Another strategy could be to attenuate their immunogenic capacity to produce inhibitors by chemically modifying them [26] or by developing recombinant factors of human origin [27].

In the long term, efforts must be directed at the development of advanced therapies, particularly strategies in the field of gene therapy (by using adeno-associated viral vectors) and cell therapy (by using adult stem cells or induced pluripotent stem cells). The chief goal of these new strategies will be to address some of the limitations associated with current treatment options such as the short in vivo half-life of administered factors, the risk of a pathogen-induced infection and the development of inhibitors. Another goal of the advanced therapies (cell therapy) will be palliative treatment of the articular consequences derived from hemophilic arthropathy [6].

## Advanced therapies for the treatment of hemophilia

In the future, the different types of advanced therapies such as gene therapy, cell therapy and tissue engineering, as well as the more recently developed induced pluripotent stem cells (iPSC) technology, may offer innumerable clinical applications for the treatment of certain monogenic diseases including hemophilia.

True as it is that hemophilia is well suited to be treated by advanced therapy protocols on account of its monogenic nature and of the fact that a modest increase in coagulation factor levels is enough to convert a severe into a moderate phenotype, it cannot be forgotten that research in this field is still at an early stage and substantial efforts will have to be made before such therapies can be made readily available to the patients, particularly in terms of safety. Safety considerations will have to be taken very seriously, notably in this group of patients who present with specific clinical characteristics that more often than not are the result of their past and present-day treatment. These characteristics include the presence of inhibitors or the predisposition to develop them, the specific immunological status of these patients and the presence of viral co-infections (HIV/HCV) [28]. For these reasons, although optimism is certainly in order, caution is of the essence to avoid raising false expectations in both physicians and patients alike.

### Gene therapy strategies

Gene therapy consists of transplantation of genetically modified cells so that they may produce a functional protein, and cell therapy in the transplantation of living cells into an organism in order to repair tissue or restore a deficient function. Both strategies are based on the use of stem cells given their indefinite capacity to renew themselves and differentiate to become cells of several specific cell lines. All these are necessary conditions for their clinical application [29].

The most significant breakthroughs in the field of advanced therapies and hemophilia are chiefly related to both preclinical and clinical trials in the fields of gene therapy (through the use of viral and non-viral vectors) and cell therapy (using several types of target cell) (Table 3). Thus hemophilia reveals itself as a disease that is highly amenable to be treated by gene therapy [30-33] by means of lentiviral and adeno-associated vectors used in adult stem cells and autologous fibroblasts, platelets or hematopoietic stem cells; and of the transfer of non-viral vectors and repair of mutations by chimeric oligonucleotides. The studies published so far have, in the most part, not reported any adverse event resulting from the application of such strategies in the clinical trials performed in terms of an immune-mediated transgene rejection (factor VIII or IX expression) although factors such as innate cellular T cell toxicity to adeno-associated capsid protein and the low efficacy obtained by non-viral vectors are impeding and limiting their success [34].

**Table 3:** Preclinical studies and clinical trials on gene- and cell therapy for hemophilia

**Authors [Reference]**	**Vector or target (tissue) cells**	**Coagulation factor expressed**	**Expression level (%)**
*Gene therapy (Preclinical studies)*			
Jeon *et al.* Ref.[35]	LVV	VIII	1-5
Brown *et al.* Ref.[34]	LVV	IX	10
Ramezani *et al.* Ref.[36]	LVV	VIII	<40
Matsui. Ref.[37]	LVV	VIII	<40
Montgomery and Shi. Ref.[40]	LVV	VIII	<40
*Gene therapy (Clinical trials)*			
Nathwani *et al.* Ref.[38]	AAV (Immunosuppressive therapy)	IX	2-11
Buchlis *et al.* Ref.[39]	AAV	IX	FIX RNA expression and AAV DNA persistence (<1% FIX)
*Cell therapy (Preclinical studies)*			
Aronovich *et al.* Ref.[43]	Embryonic day 42 spleen tissue	VIII	30-40
Follenzi *et al.* Ref.[44]	Liver sinusoidal endothelial cells	VIII	14-25
Follenzi *et al.* Ref.[45]	Kupffer cells. Bone marrow-derived mesenchymal stromal cells	VIII	10-15
Xu *et al.* Ref. [46]	iPSCs from tail-tip fibroblasts and their differentiation into endothelial cells and their precursors	VIII	8-12
Yudav *et al.* Ref. [47]	Transdifferentiation of iPSC-derived endothelial progenitor cells into hepatocytes	VIII	20

LVV, lentiviral vector; AAV, adeno-associated vector; FIX, factor IX.

Brown *et al.*[35] were the first to use lentiviral vectors for treatment of hemophilia B. Using a lentiviral vector containing a target sequence for the hematopoietic-specific microRNA, miR-142-3p, they obtained 10% factor IX activity with no anti-FIX antibodies in hemophilia B mice at over 280 days after injection. Since those results were published, use of lentiviral vectors has not ceased to grow. Thus, Jeon *et al.*[36] transduced this type of viral vector into skeletal muscle to increase factor VIII expression. Factor VIII plasma levels at one week post-injection were 5.19 ng/mL vs 0.21 ng/mL in control rats, with those levels staying constant over 4 weeks with a single dose of the vector. More recently, and also using lentiviral vectors, Ramezani *et al.*[37], adapted a nonmyeloablative conditioning regimen and directed factor VIII protein synthesis to B lineage cells using an insulated lentiviral vector containing an immunoglobulin heavy chain enhancer-promoter. Transplantation of lentiviral vector-modified hematopoietic stem cell resulted in an increase in factor VIII plasma levels for 6 months, with a low immune response against the protein expressed and a correction of the hemophilic phenotype in the transplanted mice.

Very recently, Matsui *et al.*[38], established a gene therapy strategy using autologous circulating endothelial progenitor cells transfected with lentiviral vectors containing a canine FVIII transgene. When implanted subcutaneously in a soluble basement membrane scaffold, these cells produced a long-term FVIII expression over 6 months, and resulted in effective prophylaxis against bleeding.

Ward *et al.*[39], have directly compared, using lentiviral vectors, FVIII expression from FVIII-constructs containing various B domains from non–codon-optimized and codon-optimized cDNA sequences without the confounding effect of variable immune responses against human FVIII, neo epitopes and the Fugu B domain. A dramatic increase in the observed level of secreted FVIII from codon optimized cDNA sequences was obtained. These results are in contrast to transient FVIII expression levels obtained from another many previous approaches.

Gene therapy studies conducted in hemophilic patients showed that use of adeno-associated vectors currently constitutes the most promising option given the high safety profile of such vectors, although they are not exempt from immune response-related problems. Efforts are nowadays directed at reducing the incidence of immune rejection and increasing the efficacy and length of expression. Several studies have been published in an attempt to optimize the use of viral vectors. Thus, Nathwani *et al.*[40], completed a pioneering clinical trial in patients with severe hemophilia B (<1% FIX). Patients were perfused with a dose of a serotype-8-pseudotyped, self-complementary adeno-associated vector that expressed factor IX and could efficiently transduce hepatocytes. Their results showed that factor IX expression ranged between 2 and 11% of normal values. Significant as they may seem, these results must be considered with caution as the expression levels achieved rather than normalize the patient's phenotype convert it to a mild-to-moderate form. Also treatment with glucocorticoids may be necessary to prevent immune rejection and increase the duration of transgene expression. Due account must also be taken of the fact that the adeno-associated vector has the potential to induce hepatotoxicity. For all these reasons, these undoubtedly encouraging results can only be considered a first step in the development of safe and effective advanced therapies for the treatment of hemophilia.

A recent study [41] reported on the persistent long-term expression of factor IX in parenteral administration of an adeno-associated viral vector in muscle tissue. The authors show that adeno-associated serotype-2-mediated gene transfer to human skeletal muscle persists and is transcriptionally and translationally active for a period of up to 10 years. This is the longest reported transgene expression to date.

A new alternative that has been proposed in connection with gene therapy strategies for hemophilia is the use of platelet targeting as a mechanism of drug delivery [42]. Such a strategy could play an efficient clinical role in the treatment of hemophilia and other hemostatic disorders given that it would allow local release of factor VIII and IX at the site of the bleeding-induced damage and, at the same time, protect such factors from the effect of the inhibitors potentially present in plasma.

Non-viral strategies also play an important role in this area as they constitute a safe alternative for the future in the face of the limitations that have so far been associated with viral vectors in terms of their biosafety and potential clinical application.

Thus, Sivalingam *et al.*[43], evaluated the genotoxic potential of phiC31 bacteriophage integrase-mediated transgene integration in cord-lining epithelial cells cultured from the human umbilical cord. This non-viral strategy has made it possible to obtain stable factor VIII secretion in vitro. Xenoimplantation of these protein-secreting cell lines into immunocompetent hemophilic mice corrects the severe form of the disease.

Our laboratory has advanced the use of nucleofection as a non-viral transfection method to obtain factor IX expression and secretion in adult adipose tissue-derived mesenchymal stem cells [44]. Although it is certainly true that expression efficacy with these types of protocols is lower than when viral vectors are used, it must be remembered that achieving a factor plasma level of at least 5% can transform a severe into a mild phenotype. In addition, non-viral vectors provide higher safety levels than viral ones.

### Cell therapy strategies

The use of cell therapy in the treatment of hemophilia has to date consisted mainly in the transplantation of healthy cells in an attempt to repair or replace a coagulation factor deficiency. These procedures have been conducted mainly with adult stem cells and, more recently, with progenitor cells partially differentiated from iPSCs, albeit in most cases the mechanisms by which transplanted cells (to a greater or lesser extent) engraft and go on to proliferate and function remain unknown.

Aronovich *et al.*[45], have shown that transplantation of embryonic day 42 spleen tissue in immunocompetent mice with hemophilia A attenuates the severity of the disease in the 2–3 months after the procedure. These results would seem to indicate that transplantation of a fetal spleen —obtained from a developmental stage prior to the appearance of T-cells— may potentially be used to treat some genetic disorders. For their part, Follenzi *et al.*[46], reported that once liver sinusoidal endothelial cells were transplanted and successfully engrafted into mice with hemophilia A, they were seen to proliferate and partially replace some areas of the hepatic endothelium. This resulted in a restoration of factor VIII plasma levels and in the correction of the bleeding phenotype. More recently, this same team [47] demonstrated that transplantation of healthy mouse Kupffer cells (liver macrophage/mononuclear cells), which predominantly originate from bone marrow, or of healthy bone marrow-derived mesenchymal stromal cells, can correct the phenotype of hemophilic mice and restore factor VIII levels in plasma.

As far as the use of iPSCs is concerned, the first paper was published by Xu *et al.*[48] who reported on the generation of murine iPSCs from tail-tip fibroblasts and their differentiation into endothelial cells and their precursors. These iPSC-derived cells express specific membrane markers such as CD31, CD34 and Flk1, as well as factor VIII. Following transplantation of these cells into mice with hemophilia A, the latter survived the tail-clip bleeding assay by over 3 months and their factor VIII plasma levels increased to 8%-12%. Yadav *et al.*[49], have studied the transdifferentiation of iPSC-derived endothelial progenitor cells into hepatocytes (primary cells of FVIII synthesis). These cells were injected into the liver parenchyma where they integrated functionally and made correction of the possible hemophilic phenotype. High levels of FVIII mRNA were detected in the spleen, heart, and kidney tissues of injected animals with no induction of tumors or any other adverse events in the long-term. Alipio *et al.*[50] for their part also reported on the generation of factor VIII in a hemophilic murine model one year after transplantation of iPSC-derived endothelial cells.

### Advanced therapies in the hemophilic arthropathy

Lastly, it is important to consider the potential application of advanced therapies in the palliative treatment of the articular consequences of hemophilic arthropathy. Although adequate treatment is currently available for hemophilia, which is specifically efficient regarding the negative consequences of hemophilic arthropathy, it cannot be forgotten that only 25% of hemophiliacs, most of them living in developed countries, can benefit from such treatment. In the rest of the world, hemophilic arthropathy and its disabling sequelae are the norm. But even in the developed world many patients still present moderate or severe hemophilic arthropathy on account of the fact that they either developed inhibitors or started being treated a few decades ago when present-day therapies were still unavailable.

Against this background, advanced therapies may constitute a solution of these patients [6]. Chondrocyte implantation and cell therapy using bioreactors, growth factors, mesenchymal stem cells and genetically modified cells may be used as an adjunct or even as an alternative to the current approaches (bone marrow stimulation, osteochondral autograft or allograft transplantation) for the repair of chondral damage in advanced arthropathic disease.

Mesenchymal stem cells appear hold great promise for chondral repair given their high differentiation ability and their proven therapeutic effects [51]. Implantation of autologous chondrocytes or mesenchymal stem cells was up to now able to address only highly localized chondral lesions, and the use of bioreactors and growth factors, which stimulate cartilage formation, may optimize such strategies.

## Concluding remarks

Hemophilia is highly amenable to treatment by protocols based on advanced therapies ―gene therapy, cell therapy, tissue engineering or induced pluripotent stem cell technology—. This is because it is a monogenic disease which requires low circulating levels of coagulation factor and no gene regulation to achieve a moderate phenotype, and because a large variety of pathological animal models are available for experimentation. However, advanced therapies are still at an early research phase and much effort and investment will be required before they can be applied in a generalized way. For these reasons, although optimism is warranted care must be taken not to raise false expectations in physicians or patients.

Safety is a key component in any therapeutic strategy or pharmacological product. But for patients with hemophilia safety is even more essential for several reasons including the specific clinical characteristics of the patients, such as the presence of inhibitors or the predisposition to develop them; the alteration of the individual's immunological status; or the presence of viral co-infection with HIV/HCV.

The most significant advances made in the field of advanced therapies and their application in the future treatment of several diseases, and hemophilia in particular, have been related to the development of viral gene therapy using lentiviral and adeno-associated vectors; non-viral gene transfer by nucleofection, phiC31 bacteriophage integrase or lipofection; or mutation repair by chimeric oligonucleotides. Cell therapy using adult, fetal, embryonic or IPSC-derived stem cells has also played a substantial role. Results were generally satisfactory although the plasma levels of factor VIII or IX obtained were, at best, those corresponding to a mild-moderate phenotype of the disease when viral gene transfer was employed and to a moderate phenotype when non-viral methods were used. Even if duration of expression of the protein was in some cases considerable, the factor plasma concentrations achieved were nonetheless transient and eventually cleared. Moreover, in the clinical trials carried out, hepatotoxic effects were observed as a result of the use adeno-associated vectors and of an immune response against the transgenes or the components of the virus coating themselves. Factors such as the need to use concomitant treatment with immunosuppresors to overcome some of this problems, as well as small sample sizes of patients and the difficulties inherent in extrapolation of pre-clinical results to humans and large-scale production of both vectors and cells, have so far somewhat undermined the success expected from these therapies.

Cell therapy approaches hold significant hope for palliative treatment of the disabling articular sequelae of hemophilic arthropathy in patients who, several decades ago, had no access to the optimal treatment available at present; in those that have developed inhibitors; or in that 75% of patients on the planet for whom access to adequate treatment is simply not possible.

An important consideration to be borne in mind when implementing new therapeutic strategies is the feasibility of the investment required. Given their low prevalence, treatment of many rare diseases is unfortunately not considered feasible in this respect. Feasibility of the investment must be considered in terms of justifying the long-term benefits achieved both for a certain patient population and for the entity laying out the funds. The determining factors are the number of affected individuals, the costs derived from the current treatment of these rare conditions, their chronicity and the long-term duration and their consequences (emerging pathogen-derived infections among others). The availability or otherwise of conventional treatment together with other disease-specific factors should also be taken into account.

For the reasons above, hemophilia is an excellent and financially feasible candidate for advanced therapies, if for no other reason than the high cost of its current treatment. When all of these factors are added to the fact that hemophilia is a rare low-incidence disease and to the technical and methodological problems mentioned above, it is not difficult to understand that progress will be slow. Even so, given that patient safety should always be a paramount consideration, it may be necessary to settle for slightly lower expression levels (a mild-to-moderate phenotype) in return for higher safety levels. The question is: how is this to be achieved? And, though the answer is by no means an easy one, the results obtained so far by the combination of cell therapy and gene therapy —based on viral (lentiviral and adeno-associated) vectors with improved transfection efficacy and immunogenic properties, or non-viral vectors— may be pointing in the right (and most promising) direction.

Finally, although optimism is clearly justified, fantasy is best avoided in order not to raise false expectations in the patients suffering from these rare diseases that may be subject to either curative or palliative treatment by advanced therapies protocols.

## Competing interests

AL is Principal Researcher in a preclinical project, but not a clinical trial, with adipose mesenchymal stem cells and gene/cell therapy protocols for the treatment of hemophilia. AL has no other relevant affiliations or financial involvement with any organization or entity with a financial interest in or financial conflict with the subject matter or materials discussed in this manuscript apart from those disclosed. The authors have been supported by funding from a grant from the Victoria Eugenia Royal Hemophilia Foundation.

## Authors’ contributions

AL has conceived the manuscript, and its design. CS and ASG have made intellectual contributions and have been in charge of the acquisition, analysis and interpretation of part of the literature data, being responsible for the manuscript draft and for its final revised version. All authors have read and approved this final form.

## Authors’ information

Dr. Antonio Liras is member of the Editorial Board of several scientific journals (International Archives of Medicine; Expert Review of Hematology; American Journal of Translational Research and International Journal of Clinical and Experimental Medicine). Dr. Antonio Liras is Coordinator of a project on ex vivo non-viral gene therapy and cell therapy for hemophilia using mesenchymal stem cells from human adult adipose tissue, in collaboration with the Cell Therapy and Regenerative Medicine Unit, La Paz University Hospital Health Research Institute-IdiPAZ. This project has received several awards for best research initiative in hemophilia from the Spanish Society of Thrombosis and Hemostasis, the Victoria Eugenia Royal Hemophilia Foundation and Octapharma S.A., Baxter BioScience and Pfizer Spain.
